# The Impact of Hypoglycaemia on Quality of Life in Adults With Type 1 Diabetes Using Contemporary Diabetes Technologies: A US National Survey

**DOI:** 10.1111/dom.71028

**Published:** 2026-06-25

**Authors:** Yu Kuei Lin, Uffe Søholm, Alexandria Ratzki‐Leewing, Wen Ye, Emily Hepworth, Annika Agni, Stephanie A. Amiel, Jane Speight

**Affiliations:** ^1^ Department of Internal Medicine University of Michigan Medical School Ann Arbor Michigan USA; ^2^ School of Psychology, Institute for Health Transformation Deakin University Geelong Victoria Australia; ^3^ The Australian Centre for Behavioural Research in Diabetes, Diabetes Victoria Carlton Victoria Australia; ^4^ Division of Gerontology, Department of Epidemiology and Public Health University of Maryland School of Medicine Baltimore Maryland USA; ^5^ University of Maryland Institute for Health Computing North Bethesda Maryland USA; ^6^ Department of Epidemiology and Biostatistics Schulich School of Medicine & Dentistry London Ontario Canada; ^7^ Department of Biostatistics University of Michigan School of Public Health Ann Arbor Michigan USA; ^8^ Department of Family Medicine University of Michigan Medical School Ann Arbor Michigan USA; ^9^ Department of Diabetes School of Cardiovascular and Metabolic Medicine and Sciences, King's College London London UK

**Keywords:** continuous glucose monitors, diabetes technology, hybrid closed‐loop insulin pumps, hypoglycaemia, quality of life, Type 1 diabetes

## Abstract

**Background:**

Use of contemporary diabetes technologies, including continuous glucose monitoring (CGM) and hybrid closed‐loop insulin pumps (HCLs), has expanded rapidly among people with Type 1 diabetes. However, limited research has characterised the impact of hypoglycaemia on quality of life in this population.

**Methods:**

We analysed cross‐sectional survey data from US adults aged ≥ 18 years who were using CGM or HCL and who were recruited through the T1D Exchange. Survey responses included participant characteristics, diabetes technology use, and hypoglycaemia‐specific quality of life measured by the 12‐item Hypoglycaemia Impact Profile (HIP‐12), which assesses hypoglycaemia‐related impact across 12 major life domains. HIP‐12 composite and domain‐specific scores were summarised and compared between CGM‐only (without HCL) and HCL users.

**Results:**

The analytic sample included 796 participants (mean age 47 years; 53% female), of whom 23% used CGM only and 77% used HCL. Overall, 96% reported at least one life domain negatively affected by hypoglycaemia, and 31% reported negative impacts across 10 or more of the 12 assessed domains. The most frequently negatively affected domains were *sleep*, *leisure activities*, *emotional well‐being*, *spontaneity* and *physical activity/fitness*. Compared with CGM‐only users, HCL users reported greater hypoglycaemia‐related negative impacts on *leisure activities* (*p* < 0.001), *physical activity/fitness* (*p* < 0.001), *spontaneity* (*p* = 0.011), *sex life* (*p* = 0.014) and *work or studies* (*p* = 0.021).

**Conclusions:**

Hypoglycaemia continues to adversely affect multiple related quality‐of‐life domains despite using contemporary diabetes technologies. Routine clinical assessment of and clinical support for hypoglycaemia's impact remain important. Further research is needed to clarify the mechanisms underlying these impacts and to guide targeted interventions.

## 1 Introduction

Hypoglycaemia is a major complication of insulin therapy experienced by people with Type 1 diabetes [1, 2]. Each hypoglycaemic episode can trigger a range of unpleasant symptoms, including shakiness, irritability, confusion, palpitations, sweating and hunger [3], that disrupt daily functioning [4] and social interactions [5]. The potential for hypoglycaemia to lead to serious physical health consequences, including cardiac arrhythmias [6], injuries from motor vehicle accidents [7] and death [8], can engender substantial fear that impedes optimal self‐management behaviour [9]. Hypoglycaemia also places a burden on family members and caregivers [10] and contributes to financial consequences through treatment costs [11], reduced work productivity, absenteeism or lateness [12] and medical expenses [13]. The associated decline in health utility is thus comparable to that observed with major diabetes‐related conditions such as angina pectoris and renal failure [14]. Consequently, despite advances in diabetes care, hypoglycaemia remains one of the most important concerns identified by people living with Type 1 diabetes [15].

Prior research has demonstrated that generic and diabetes‐specific quality‐of‐life measures lack the specificity to capture hypoglycaemia's distinct impacts [5], which may explain inconsistent findings regarding the relationship between hypoglycaemia and overall diabetes‐related quality of life [16]. To address this limitation, the 12‐item Hypoglycaemia Impact Profile (HIP‐12) was developed and validated to assess hypoglycaemia‐related impacts across 12 life domains [17]. A prior study using this instrument has shown that reduced hypoglycaemia‐related quality of life is common and a major concern among adults with Type 1 diabetes [18].

While contemporary diabetes technologies, including continuous glucose monitoring (CGM) and hybrid closed‐loop insulin delivery systems (HCL), can improve psychological outcomes (such as diabetes distress and fear of hypoglycaemia [19]), they may also introduce new burdens [20], including frequent alarms, device management demands and unwanted attention from others during hypoglycaemia episodes [21]. However, hypoglycaemia‐specific quality of life has not been well characterised in populations with high uptake of these technologies. Moreover, no prior studies have directly compared hypoglycaemia‐related quality of life across subgroups defined by diabetes technology use.

As standard practice continues to embrace CGM and HCL use [22], a comprehensive understanding of hypoglycaemia‐specific quality of life in the context of these technologies is needed. Such knowledge is essential to inform targeted support strategies and optimise care delivery. This study, thus, aimed to (1) evaluate hypoglycaemia‐specific quality of life in a nationally recruited US cohort of adults with Type 1 diabetes and high uptake of CGM and HCL and (2) characterise domain‐specific impacts across subgroups defined by technology use.

## 2 Materials and Methods

### 2.1 Study Design, Setting, Participants and Ethics

Our research team conducted an online cross‐sectional survey among registrants of the T1D Exchange, a US National Type 1 Diabetes Registry, between September 2024 and February 2025. This survey represents the second‐year follow‐up of a larger longitudinal observational study conducted by our research team [23] that enrolled adults aged 18 years or older with Type 1 diabetes to track self‐reported hypoglycaemia awareness over a 3‐year period (2023–2025).

Invitations were emailed to all 1580 participants in the parent study, with up to three contact attempts, including reminder emails and telephone calls. Enrolment continued until responses were received from all parent study participants or until 4 months had elapsed, whichever occurred first.

The study was approved by the University of Michigan Institutional Review Board (HUM#00232351). All participants provided informed consent before survey initiation.

### 2.2 Measures

The survey captured self‐reported demographic characteristics, including age, gender, race, ethnicity, education level and annual household income, as well as clinical characteristics, including diabetes duration, current status of the use of CGM with and without HCL, and self‐reported haemoglobin A1C and history of severe hypoglycaemia in the past 6 months (defined as any episode requiring assistance from another person for recovery [24]). Hypoglycaemia awareness was assessed using the impaired awareness subscale of the Hypoglycaemia Awareness Questionnaire [25], with a composite score ≥ 12 indicating impaired awareness of hypoglycaemia (IAH) [26].

Hypoglycaemia‐related quality of life was measured using the HIP‐12, a validated instrument for adults with Type 1 diabetes [17]. The HIP‐12 includes 12 items, each corresponding to a distinct life domain: physical health, financial situation, relationships, leisure activities, work or studies, emotional well‐being, sleep, dietary freedom, sex life, independence, spontaneity and physical activity/fitness. Each item is rated on a 7‐point Likert scale ranging from *very positive impact* (Score 1) to *neutral* (Score 4) to *very negative impact* (Score 7), with a *not applicable* option available for each domain.

### 2.3 Statistical Analysis

Only participants who completed all survey items, including the HIP‐12 questionnaire, were included in the analysis. Because only a small number of participants reported using neither CGM nor HCL (*n* = 22), and this subgroup was considered insufficient for meaningful comparison, results reporting is restricted to participants using CGM without HCL and those using HCL.

Participant characteristics were summarised using descriptive statistics. The HIP‐12 composite score was calculated as the mean of all item responses for participants who provided scorable responses (i.e., did not select *not applicable*) for at least seven domains [17]; higher composite scores indicated greater negative impact of hypoglycaemia on overall quality of life. For domain‐level analyses, responses marked as *not applicable* were also excluded. Continuous and categorical variables were compared using the Mann–Whitney *U* test and chi‐square test, respectively. All analyses were conducted using IBM SPSS Statistics (version 31.0). A two‐sided *p* < 0.05 was considered statistically significant.

## 3 Results

### 3.1 Participant Characteristics

A total of 843 participants initiated the survey; of these individuals, 25 were excluded due to incomplete responses and 22 due to non‐use of CGM or HCL. Characteristics of the remaining 796 participants are summarised in Table 1. The mean (SD) age was 47 (16) years, diabetes duration 27 (16) years, and HbA1c 6.6 (0.9)%. Overall, 419 (53%) participants self‐identified as women, 744 (93%) as White, and 744 (93%) as non‐Hispanic. Regarding diabetes technology use, 183 (23%) used CGM without HCL, and 613 (77%) used HCL.

**TABLE 1:** Participant characteristics across the overall cohort and by technology use subgroup.

	All participants (*N* = 796)	CGM user without HCL (*n* = 183)	HCL users (*n* = 613)
Age (years)	47 (16)	51 (17)	46 (15)
Gender			
Male	365 (46)	101 (55)	264 (43)
Female	419 (53)	80 (43)	339 (55)
Other	12 (2)	2 (1)	10 (2)
Race			
White	744 (93)	164 (90)	580 (95)
Other	52 (7)	19 (10)	33 (5)
Ethnicity			
Non‐Hispanic	744 (93)	172 (94)	572 (93)
Hispanic	52 (7)	11 (6)	41 (7)
Education level			
Associate degree or below	218 (27)	55 (30)	163 (27)
Bachelor's degree	317 (40)	76 (42)	241 (39)
Master's degree or above	259 (32)	52 (28)	207 (34)
Do not wish to provide	2 (< 1)	0 (0)	2 (< 1)
Household income			
$50 000 or below	170 (21)	49 (27)	121 (20)
$50 000–< $100 000	227 (29)	54 (30)	173 (28)
$100 000–< $200 000	244 (31)	47 (26)	197 (32)
$200 000 or above	90 (11)	16 (9)	74 (12)
Do not wish to provide/don't know	65 (8)	17 (9)	48 (8)
Diabetes duration (years)	27 (16)	26 (17)	27 (16)
HbA1c (%)			
%	6.6 (0.9)	6.8 (1.2)	6.6 (0.8)
mmol/mol	49 (11)	51 (13)	49 (8)
CGM use duration, year (*n* = 796)	7.5 (5.6)	5.6 (3.9)	8.1 (5.6)
Severe hypoglycaemia incidence in the past 6 months	1.0 (5.0)	1.2 (4.0)	0.9 (5.2)
At least one severe hypoglycaemia	171 (21)	47 (27)	124 (20)
HypoA‐Q Impaired Awareness Subscale	7.1 (3.9)	7.0 (4.0)	7.2 (3.9)
Impaired awareness of hypoglycaemia	108 (14)	23 (13)	85 (14)

*Note:* Data presented in *n* (%) or mean (standard deviation).

Abbreviations: CGM, continuous glucose monitoring; HbA1c, haemoglobin A1C; HCL, hybrid closed‐loop insulin pump; HIP‐12, 12‐item Hypoglycaemia Impact Profile; HypoA‐Q, Hypoglycaemia Awareness Questionnaire.

^a^ *n* = 815 for all participants, *n* = 22 for non‐technology users.

HIP‐12 composite scores were available for 793 (99.6%) participants (Table 2). The mean (SD) HIP‐12 composite score was 4.9 (0.7), indicating a slightly negative overall impact of hypoglycaemia on quality of life across the cohort.

**TABLE 2:** Hypoglycaemia impact on quality of life by diabetes technology use.

	All participants (*N* = 796)	CGM‐only users (*n* = 183)	HCL users (*n* = 613)	*p* ^a^
HIP‐12 composite score (*n* = 793^b^)	4.9 (0.7)	4.8 (0.9)	4.9 (0.7)	**0.022**
Physical health (*n* = 790)	4.8 (1.1)	4.7 (1.2)	4.8 (1.0)	0.345
Financial situation (*n* = 775)	4.5 (0.9)	4.6 (1.1)	4.5 (0.9)	0.104
Relationships (*n* = 786)	4.5 (0.9)	4.5 (1.0)	4.5 (0.9)	0.332
Leisure activities (*n* = 788)	5.0 (1.0)	4.8 (1.1)	5.1 (1.0)	**< 0.001**
Work or studies (*n* = 743)	4.8 (1.0)	4.7 (1.1)	4.9 (0.9)	**0.021**
Emotional well‐being (*n* = 792)	5.1 (1.1)	5.0 (1.2)	5.1 (1.1)	0.390
Sleep (*n* = 794)	5.3 (1.1)	5.4 (1.3)	5.3 (1.0)	0.317
Dietary freedom (*n* = 783)	5.0 (1.3)	5.0 (1.3)	5.0 (1.3)	0.800
Sex life (*n* = 714)	4.8 (1.0)	4.6 (1.0)	4.8 (1.0)	**0.014**
Independence (*n* = 787)	4.7 (1.0)	4.6 (1.2)	4.7 (1.0)	0.337
Spontaneity (*n* = 793)	5.2 (1.1)	5.0 (1.2)	5.2 (1.1)	**0.011**
Physical activity/fitness (*n* = 791)	5.0 (1.1)	4.6 (1.2)	5.1 (1.1)	**< 0.001**

*Note:* Data presented as mean (SD).

Abbreviations: CGM, continuous glucose monitoring; HCL, hybrid closed‐loop insulin pump.

^a^ Mann–Whitney *U* tests were performed for pairwise comparison between CGM‐only and HCL users; bolded if statistically significant.

^b^ *n* = 182 for CGM users without HCL, *n* = 611 for HCL users who provided scorable responses (i.e., did not select not applicable) for at least seven domains.

### 3.2 Patterns of Hypoglycaemia Impact Across Life Domains

**FIGURE 1:**
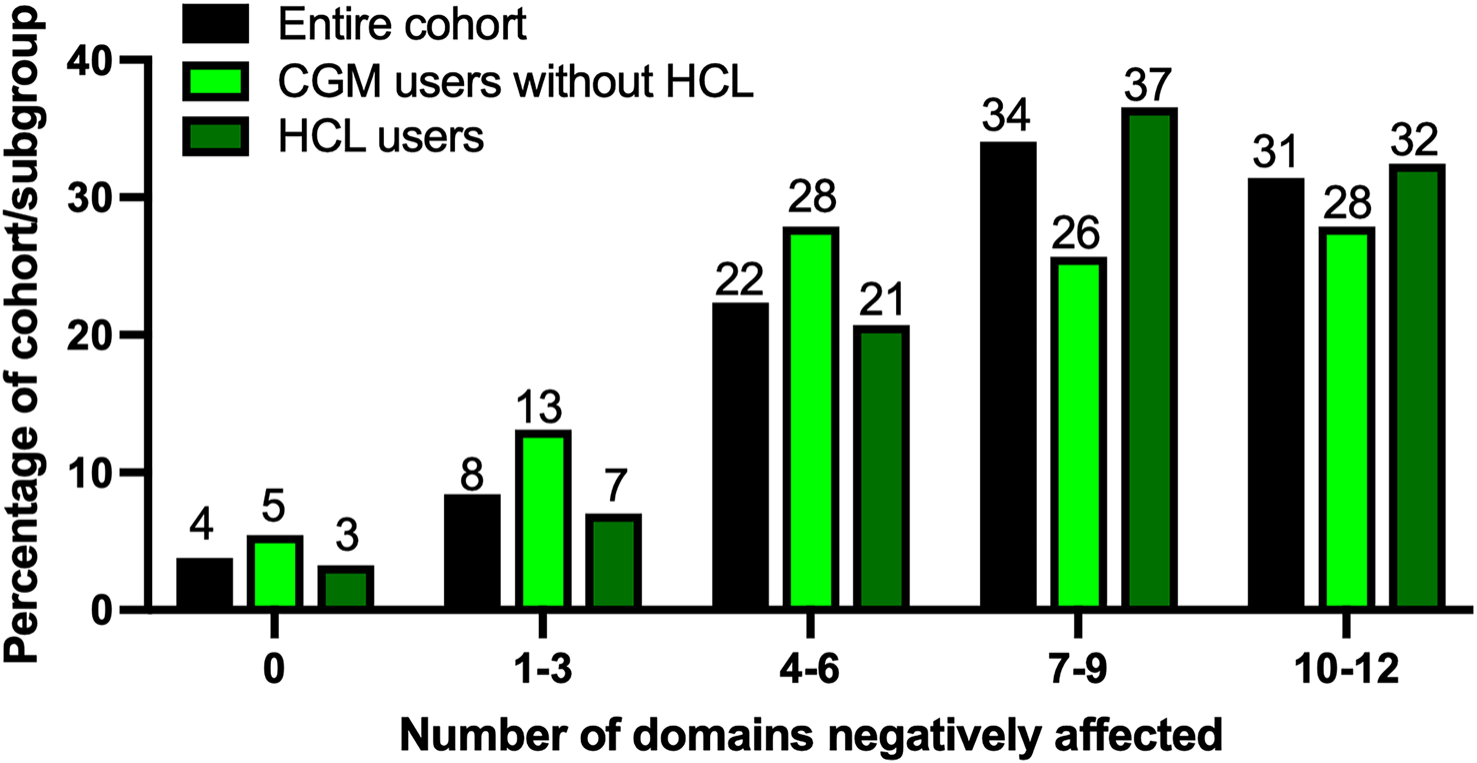
Percentage of participants reporting the number of domains negatively affected by hypoglycaemia in the overall cohort and diabetes technology subgroups. CGM, continuous glucose monitoring; HCL, hybrid closed‐loop insulin pump.

**FIGURE 2:**
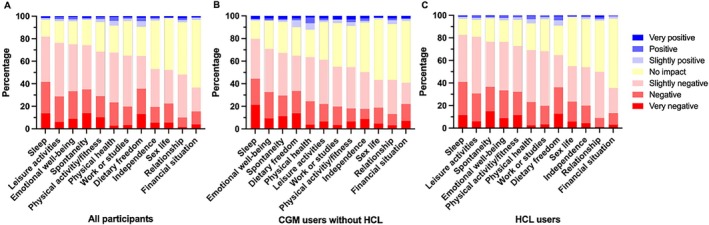
Percentage of participants reporting the impact of hypoglycaemia, illustrated for (A) all participants, (B) CGM users without HCL and (C) HCL users. Domains are ranked by the percentage of participants reporting a negative impact on their lives. CGM, continuous glucose monitoring; HCL, hybrid closed‐loop insulin pump.

In the overall cohort, 96% of participants reported at least one life domain negatively affected by hypoglycaemia, while nearly one‐third (31%) reported negative impacts across 10 or more of the 12 assessed domains (Figure 1). More than half of participants reported negative impacts in 10 of the 12 life domains (Figure 2), with *sleep* (82%), *leisure activities* (77%), *emotional well‐being* (75%), *spontaneity* (74%) and *physical activity/fitness* (68%) most frequently affected.

Among the subgroups, 95% of CGM users without HCL and 97% of HCL users reported at least one negatively affected domain, while 28% of CGM users without HCL and 32% of HCL users reported negative impacts across 10 or more of the 12 domains (Figure 1). More than half of participants reported negative impacts across 9 domains among CGM users without HCL and 10 domains among HCL users (Figure 2). In both subgroups, *sleep*, *emotional well‐being* and *spontaneity* were consistently ranked among the five most frequently affected domains.

Approximately half of participants who reported negative impacts on *sleep*, emotional well‐being, *spontaneity* or *dietary freedom* rated these effects as *moderate* or *severe* in both subgroups (Table S1). Although fewer participants reported negative impacts on *sex life*, nearly half of those affected rated these impacts as *moderate* or *severe* in both subgroups.

### 3.3 Comparative Impact of Hypoglycaemia Across Diabetes Technology Subgroups

**FIGURE 3:**
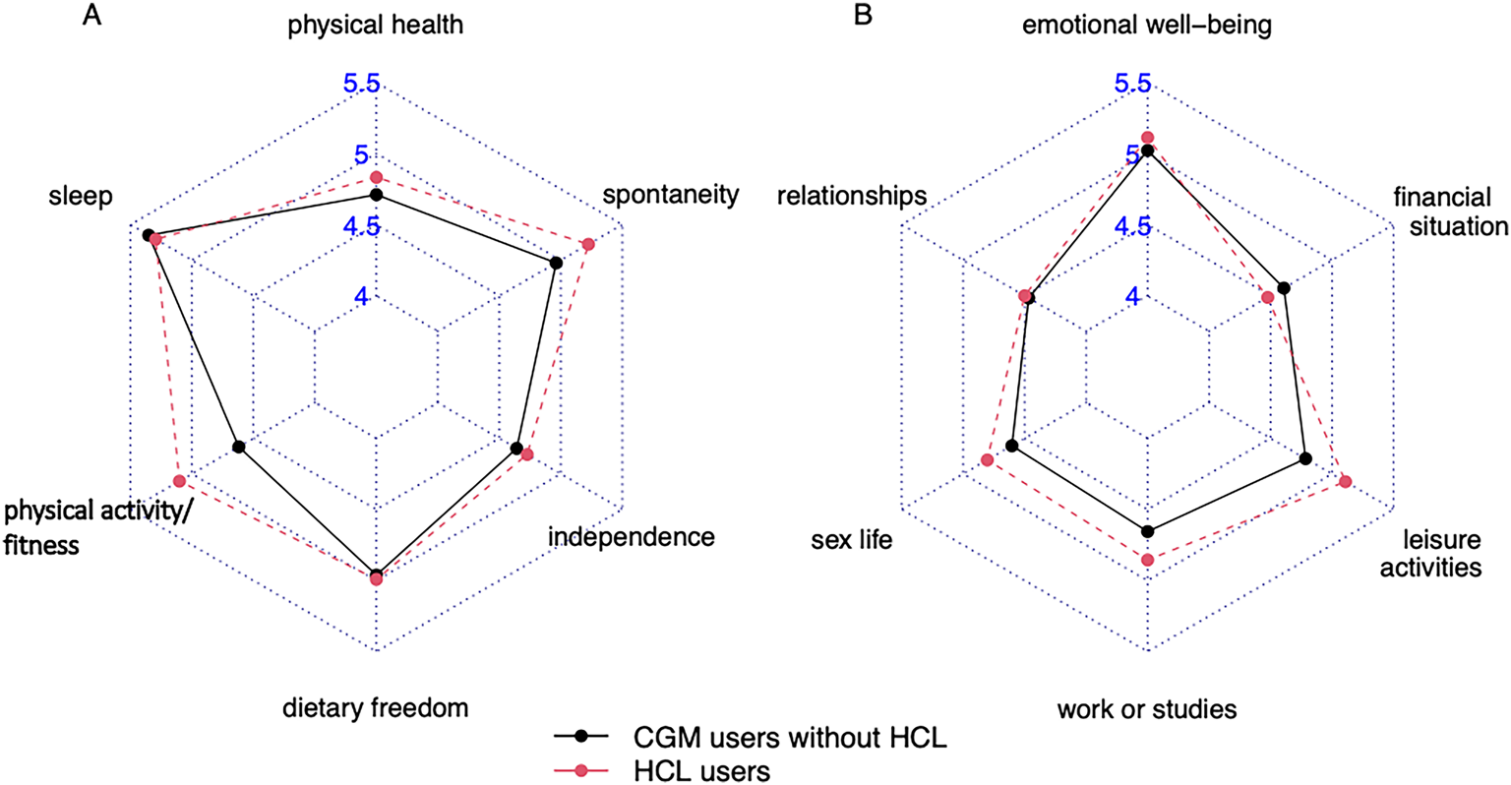
Radar plots of mean HIP‐12 domain scores categorised by (A) health and functioning and (B) life participation and fulfilment. CGM, continuous glucose monitoring; HCL, hybrid closed‐loop insulin pump; HIP‐12, 12‐item Hypoglycaemia Impact Profile.

HCL users had higher composite scores than CGM users without HCL (*p* = 0.022). Although mean scores were similar across most life domains, differences were observed in several domains among the two technology subgroups (Figure 3). In statistical analyses, HCL users reported a greater hypoglycaemia‐related negative impact than CGM users without HCL for *leisure activities* (*p* < 0.001), *physical activity/fitness* (*p* < 0.001), *sex life* (*p* = 0.014), *spontaneity* (*p* = 0.011) and *work or studies* (*p* = 0.021) (Table 2).

## 4 Discussion

In this nationally recruited US cohort of adults with Type 1 diabetes using contemporary diabetes technologies, hypoglycaemia negatively affects quality of life, most notably in the domains of *sleep*, *leisure activities*, *emotional well‐being*, *spontaneity* and *physical activity/fitness*, with nearly one‐third of people reporting negative impacts across most or all assessed life domains. Although the overall pattern of hypoglycaemia‐related impact was broadly similar between CGM users without HCL and HCL users, HCL users reported greater negative impacts in *leisure activities*, *physical activity/fitness*, *sex life*, *spontaneity* and *work or studies*.

This study extends prior work by evaluating hypoglycaemia‐specific quality of life in a population with high uptake of and sustained use of contemporary diabetes technologies. Three findings merit emphasis. First, hypoglycaemia continues to adversely affect quality of life even among users of advanced technologies; clinicians should therefore not assume that hypoglycaemia‐related burdens are minimal in this population. Second, hypoglycaemia impacts are often domain‐specific [5, 18]. Although composite HIP‐12 scores may suggest a relatively modest overall burden, some individuals experience substantial negative impacts in particular domains, underscoring the importance of domain‐level assessment. Third, the perceived impact of hypoglycaemia varied by technology subgroup, generating hypotheses regarding how patient characteristics, technology use, and hypoglycaemia experiences jointly influence quality of life [27, 28].

Compared with prior data from cohorts with lower uptake of contemporary diabetes technologies [18], participants in our cohort appeared to report lower hypoglycaemia‐related negative impacts in several domains, including *physical health*, *leisure activities*, *work or studies* and *independence*. These findings may be consistent with the potential benefits of advanced diabetes technologies in mitigating the day‐to‐day burden of hypoglycaemia. However, participants in our cohort also reported greater negative impacts in the domains of *sex life* and *financial situation*, resulting in overall HIP‐12 composite scores that were comparable to those reported previously [18]. Device‐related factors may help explain the greater negative impacts observed in these two domains among technology users. For example, CGM and HCL may disrupt intimate activities through alarms or the need for device management during periods of actual or anticipated hypoglycaemia. In addition, although CGM can facilitate the prevention and management of hypoglycaemia, greater use of hypoglycaemia treatments may increase out‐of‐pocket expenses, potentially contributing to perceived financial burden [11].

Moreover, although in domains such as *sleep*, *dietary freedom* and *physical activities*, the hypoglycaemia‐related impact in our participants was comparable to that observed in the prior cohort with lower technology uptake [18], the underlying mechanisms may differ. Among individuals not using diabetes technologies, nocturnal hypoglycaemia symptoms (e.g., nightmares or post‐episode arousal [29]) may predominate, whereas among CGM and HCL users, alarms and device management may contribute more substantially [30]. In addition, while CGM enables early detection and prevention of hypoglycaemia, concerns regarding data accuracy issues, anticipatory management, and device‐related tasks may themselves contribute to perceived burden across multiple life domains [30]. Future research incorporating specific measures of device reliability, usability, and user satisfaction may provide further insight into how diabetes technologies influence hypoglycaemia‐related quality of life.

The greater overall and domain‐specific negative impact of hypoglycaemia observed among HCL users, particularly in domains such as *spontaneity*, compared with CGM‐only users, is less straightforward to interpret. One possible explanation is confounding by indication: individuals who initiate HCL may have experienced greater baseline disruption, with HCL reducing, but not eliminating, this burden [19]. Alternatively, technology‐related demands (e.g., alerts, troubleshooting and planning around devices) may reduce perceived spontaneity despite improved glycaemic control [31]. Finally, although HCL users in our cohort experienced less severe hypoglycaemia than CGM users without HCL, those with inappropriate HCL settings may have had more frequent day‐to‐day hypoglycaemia and greater associated negative impacts.

Consistent with prior work [18, 32], a small subset of participants reported positive impacts of hypoglycaemia. Qualitative studies suggest that such perceptions may arise in contexts of increased social support or strengthened interpersonal relationships [21, 33]. Future research should clarify the contexts in which positive perceptions emerge, their neurobiological correlates [34], and whether they may impede appropriate hypoglycaemia self‐management [35] while mitigating diabetes‐related burnout [36].

Although contemporary diabetes technologies can improve glycaemic outcomes, including reducing hypoglycaemia [37, 38], real‐world studies suggest that hypoglycaemia continues to occur despite these technologies. Given that prospective users may hold high expectations regarding these technologies' ability to reduce the burden of diabetes [39], both the benefits and limitations of these technologies across different life domains should be clearly discussed to minimise mismatches between expectations and real‐world experiences. Our findings further suggest that active screening for the quality‐of‐life impacts of hypoglycaemia remains important, even among individuals with sustained use of advanced technologies. Such assessments may help identify domain‐specific burdens and inform education and support needs, helping individuals navigate both hypoglycaemia‐related challenges and burdens associated with technology use.

Strengths of this study include national recruitment, enhancing geographic diversity; a high average duration of CGM use, enabling evaluation of hypoglycaemia‐specific quality of life among individuals with longitudinal experiences using contemporary technologies; and the use of a validated, multidomain instrument to assess hypoglycaemia's impact on quality of life. Limitations include recruitment from a parent study composed primarily of non‐Hispanic, well‐educated individuals with relatively well‐controlled HbA1c, a higher rate of severe hypoglycaemia, and interest in research, which reflect reporting bias and may limit generalisability. As with many national survey‐based studies, the absence of third‐party verification may limit the accuracy of self‐reported information. We also did not recruit a sufficient number of non‐technology users to conduct additional subgroup comparisons. Finally, the cross‐sectional design and between‐group differences preclude causal inference regarding the effects of technology use on quality‐of‐life outcomes.

In conclusion, hypoglycaemia continues to adversely affect hypoglycaemia‐specific quality of life even in the context of contemporary diabetes technologies, with nearly one‐third of technology users reporting negative impact across almost all life domains. Clinicians should not assume that technology users experience minimal burden; rather, detailed, domain‐specific assessment is needed to guide targeted support. Education for prospective users of advanced diabetes technologies should include discussions of the technologies' limitations and their potential impact during hypoglycaemic events. Future research should comprehensively evaluate how patient characteristics, technology use and hypoglycaemia experiences, both actual and anticipatory, jointly influence hypoglycaemia‐specific quality of life, including disentangling the direct effects of hypoglycaemia from the burdens associated with device use.

## Author Contributions

Y.K.L., U.S., W.Y., A.R.‐L., S.A.A. and J.S. were involved in the conception, design, and interpretation of the results. E.H. and A.A. collected the data. Y.K.L. and W.Y. conducted the analyses. Y.K.L. wrote the first draft of the manuscript, and all authors edited, reviewed and approved the final version of the manuscript.

## Funding

This work was supported by the Michigan Center for Clinical and Translational Research (P30DK092926) and Michigan Diabetes Research Center, University of Michigan (P30DK020572). REDCap was supported by the National Center for Advancing Translational Sciences (UM1TR004404). Y.K.L. was supported by the National Institute of Diabetes and Digestive and Kidney Diseases (K23DK129724 and R01DK142830). U.S. and J.S. were supported by core funding to the Australian Centre for Behavioural Research in Diabetes provided by the collaboration between Diabetes Victoria and Deakin University.

## Conflicts of Interest

Alexandria Ratzki‐Leewing reports research support from Sanofi Global and Sanofi Canada; consulting for Vertex, Dexcom Canada, Abbott Canada, and Sanofi United States; advisory boards for Sanofi United States; and paid presentations for Sanofi Canada, Sanofi Global, Abbott Canada and Dexcom Canada. Yu Kuei Lin reports research support from DEKA Research and Development. Uffe Søholm has previously been employed by Novo Nordisk A/S. Jane Speight has served on advisory boards for Janssen, Medtronic, Omnipod, Roche Diabetes Care and Sanofi Diabetes; received unrestricted educational grants and in‐kind support from Abbott Diabetes Care, AstraZeneca, Medtronic, Roche Diabetes Care and Sanofi Diabetes; received sponsorship to attend educational meetings from Medtronic, Roche Diabetes Care and Sanofi Diabetes and consultancy income or speaker fees from Abbott Diabetes Care, AstraZeneca, Insulet, Medtronic, Novo Nordisk, Roche Diabetes Care, Sanofi Diabetes and Vertex. In all cases, Jane Speight's research group has been the beneficiary. The other authors declare no conflicts of interest.

## Supporting information


**Table S1:** Domain‐specific hypoglycaemia impact and affected participant percentages.

## Data Availability

The quality of life data that support the findings of this study are available in the Supporting Information of this article. Additional participant characteristics data are available on request from the corresponding author.
