# Supplementary material for: The Impact of Hypoglycaemia on Quality of Life in Adults With Type 1 Diabetes Using Contemporary Diabetes Technologies: A US National Survey
Source: Diabetes Obes Metab. 2026 Jun 25;28(9):8390–7. doi: 10.1111/dom.71028 (PMC13332710; doi:10.1111/dom.71028)
Supplement: Supplementary file 1 [file DOM-28-8390-s001.docx]

**Supplemental Table 1. Domain-Specific Hypoglycemia Impact and Affected Participant Percentages**

|  | *Very positive* | *Positive* | *Slightly positive* | *No impact* | *Slightly negative* | *Negative* | *Very negative* |
| --- | --- | --- | --- | --- | --- | --- | --- |
| **Overall cohort (n=796)** | | | | | | | |
| Physical health (n=790) | 7 (1) | 26 (3) | 33 (4) | 189 (24) | 350 (44) | 163 (21) | 22 (3) |
| Financial situation (n=775) | 5 (1) | 6 (1) | 15 (2) | 465 (60) | 164 (21) | 89 (11) | 31 (4) |
| Relationships (n=786) | 15 (2) | 12 (2) | 15 (2) | 365 (46) | 300 (38) | 66 (8) | 13 (2) |
| Leisure activities (n=788) | 7 (1) | 14 (2) | 13 (2) | 152 (19) | 375 (48) | 179 (23) | 48 (6) |
| Work or studies (n=743) | 7 (1) | 9 (1) | 15 (2) | 229 (31) | 336 (45) | 122 (16) | 25 (3) |
| Emotional well-being (n=792) | 7 (1) | 15 (2) | 13 (2) | 163 (21) | 330 (42) | 193 (24) | 71 (9) |
| Sleep (n=794) | 12 (2) | 3 (0) | 12 (2) | 117 (15) | 318 (40) | 222 (28) | 110 (14) |
| Dietary freedom (n=783) | 10 (1) | 24 (3) | 39 (5) | 204 (26) | 227 (29) | 176 (22) | 103 (13) |
| Sex life (n=714) | 8 (1) | 2 (0) | 5 (1) | 326 (46) | 212 (30) | 121 (17) | 40 (6) |
| Independence (n=787) | 9 (1) | 9 (1) | 13 (2) | 338 (43) | 265 (34) | 110 (14) | 43 (5) |
| Spontaneity (n=793) | 10 (1) | 8 (1) | 9 (1) | 177 (22) | 311 (39) | 167 (21) | 111 (14) |
| Physical activity/fitness (n=791) | 8 (1) | 13 (2) | 20 (3) | 208 (26) | 312 (39) | 148 (19) | 82 (10) |
| **CGM users without HCL (n=183)** | | | | | | | |
| Physical health (n=180) | 3 (2) | 9 (5) | 10 (6) | 44 (24) | 70 (39) | 37 (21) | 7 (4) |
| Financial situation (n=181) | 4 (2) | 1 (1) | 3 (2) | 99 (55) | 34 (19) | 27 (15) | 13 (7) |
| Relationships (n=180) | 4 (2) | 3 (2) | 6 (3) | 89 (49) | 54 (30) | 18 (10) | 6 (3) |
| Leisure activities (n=181) | 4 (2) | 3 (2) | 3 (2) | 60 (33) | 71 (39) | 28 (15) | 12 (7) |
| Work or studies (n=171) | 5 (3) | 1 (1) | 5 (3) | 66 (39) | 60 (35) | 28 (16) | 6 (4) |
| Emotional well-being (n=181) | 4 (2) | 3 (2) | 1 (1) | 45 (25) | 69 (38) | 42 (23) | 17 (9) |
| Sleep (n=182) | 5 (3) | 1 (1) | 2 (1) | 29 (16) | 64 (35) | 42 (23) | 39 (21) |
| Dietary freedom (n=179) | 4 (2) | 3 (2) | 11 (6) | 45 (25) | 56 (31) | 35 (20) | 25 (14) |
| Sex life (n=164) | 3 (2) | 0 (0) | 2 (1) | 88 (54) | 40 (24) | 23 (14) | 8 (5) |
| Independence (n=181) | 4 (2) | 4 (2) | 3 (2) | 79 (44) | 59 (33) | 16 (9) | 16 (9) |
| Spontaneity (n=183) | 5 (3) | 2 (1) | 3 (2) | 50 (27) | 69 (38) | 33 (18) | 21 (11) |
| Physical activity/fitness (n=181) | 6 (3) | 4 (2) | 6 (3) | 66 (36) | 66 (36) | 21 (12) | 12 (7) |
| **HCL users (n=613)** | | | | | | | |
| Physical health (n=610) | 4 (1) | 17 (3) | 23 (4) | 145 (24) | 280 (46) | 126 (21) | 15 (2) |
| Financial situation (n=594) | 1 (0) | 5 (1) | 12 (2) | 366 (62) | 130 (22) | 62 (10) | 18 (3) |
| Relationships (n=606) | 11 (2) | 9 (1) | 9 (1) | 276 (46) | 246 (41) | 48 (8) | 7 (1) |
| Leisure activities (n=607) | 3 (0) | 11 (2) | 10 (2) | 92 (15) | 304 (50) | 151 (25) | 36 (6) |
| Work or studies (n=572) | 2 (0) | 8 (1) | 10 (2) | 163 (28) | 276 (48) | 94 (16) | 19 (3) |
| Emotional well-being (n=611) | 3 (0) | 12 (2) | 12 (2) | 118 (19) | 261 (43) | 151 (25) | 54 (9) |
| Sleep (n=612) | 7 (1) | 2 (0) | 10 (2) | 88 (14) | 254 (42) | 180 (29) | 71 (12) |
| Dietary freedom (n=604) | 6 (1) | 21 (3) | 28 (5) | 159 (26) | 171 (28) | 141 (23) | 78 (13) |
| Sex life (n=550) | 5 (1) | 2 (0) | 3 (1) | 238 (43) | 172 (31) | 98 (18) | 32 (6) |
| Independence (n=606) | 5 (1) | 5 (1) | 10 (2) | 259 (43) | 206 (34) | 94 (16) | 27 (4) |
| Spontaneity (n=610) | 5 (1) | 6 (1) | 6 (1) | 127 (21) | 242 (40) | 134 (22) | 90 (15) |
| Physical activity/fitness (n=610) | 2 (0) | 9 (1) | 14 (2) | 142 (23) | 246 (40) | 127 (21) | 70 (11) |

Data presented in participant count (%). CGM, continuous glucose monitoring; HCL, hybrid closed-loop insulin pump.
